# Time is of the essence in headache measurement. A response to Gil-Gouveia

**DOI:** 10.1186/s10194-022-01404-0

**Published:** 2022-03-05

**Authors:** TJ. Steiner

**Affiliations:** 1 Department of Neuromedicine and Movement Science, NTNU Norwegian University of Science and Technology, Edvard Griegs gate, Trondheim, Norway; 2 Division of Brain Sciences, Imperial College London, London, UK

Cost-effectiveness analysis of structured headache services, proposed as the health-care solution to headache [1], required a new outcome measure, universal in the sense of being applicable to multiple headache types and to all forms of treatment, care and care-delivery systems as opposed to comparisons of single-modality treatments. Ideally this measure would be expressed in intuitive units [2]. *Pain* is the principal symptom of most headache disorders (particularly those that are common: migraine, tension-type headache [TTH] and medication-overuse headache [MOH]), but difficult to value in economic terms. *Time*, on the other hand, is readily measured. Thus, a meaningful measure of headache can be based on time in ictal state (TIS), either as total TIS or as a proportion of all time (pTIS) [2]. Acute treatments diminish TIS (and pTIS) by shortening attack duration, preventative treatments by reducing attack frequency; health-care systems such as structured headache services deliver these benefits by expanding the reach and coverage of these treatments [1, 2].

How might this work as a headache metric? By factoring in the disability weight (DW) for the ictal state (of migraine, TTH or MOH) supplied by the Global Burden of Disease (GBD) studies [3], the impact of any of these headache disorders on health is expressed as TIS*DW [2]. If the time unit is hours, TIS*DW yields hours lived with disability (HLDs), in analogy with GBD’s years lived with disability (YLDs) [3]. (It should be noted that, despite their name, DWs are estimates of *lost health* rather than disability [4, 5].) In the universal outcome measure developed by Steiner et al., the benefits from treatment, of whatever type and however provided, are all measurable as *HLDs-averted* [2]. Population-level estimates factor in prevalence, treatment coverage and adherence. For health-care systems, gains from provider-training (promoting adherence to guidelines and thereby enhancing coverage) and from consumer-education (improving adherence to care plans), both increasing numbers within populations receiving the benefits of treatments, are measurable by the same metric [2].

Gil-Gouveia’s Headache Gauge (HG) extends the concept of a *time*-based outcome measure to routine clinical practice and clinical trials [6, 7]. HG is essentially a product of TIS and disability [7]. It is calculated as “daily impact scores” (labelled *s*_*i*_), which are summed over a period of time and expressed as a proportion of the maximum possible score [7]. Since both TIS and disability are assessed on four-point ordinal scales, which are then treated as interval scales 1–4 (more about this later), the maximum score is 16 N, where N is the number of days of observation.

HG is a reincarnation of the old "Headache index" (frequency * duration * intensity) as a measure of headache. This fell into disfavour when IHS produced its first guidelines for clinical trials in migraine [8] because of the uncertainties in duration estimation (often obscured by sleep), the insensitivity and subjectivity of the intensity scale 1–3, and the questionable independence of intensity and duration (severe attacks take longer to resolve). HG seeks to avoid the first of these by prospective daily assessment (eliminating recall error), and the second and third by replacing intensity with a disability estimate, directly and contemporaneously reported by the person affected. Conceptually, this makes sense: pain is disabling, which for most people is the consequence of headache that is of greatest concern. Nonetheless, in the development of HG, attack frequency and duration were noted to be its main drivers [7]. Since these are the two factors of TIS, HG reflects *time* as a casualty of headache more than the disability it is estimated to cause.

How real is this finding? On the one hand, HG imports disability imprecisely: as 1–4, where 1 = “allows full function”, 2 = “interferes with normal activities”, 3 = “impedes normal activities” and 4 = “bedridden or hospitalized” [7]. On the other, a recent analysis correlated lost productive time attributed to migraine with (pTIS*DW) [10], the former measured using the Headache-Attributed Lost Time (HALT) index [9], the latter essentially a time measure since the DW for migraine is a constant (0.441 [3]). While a linear relationship was demonstrated, it was not so strong as might be expected, being much influenced by many extraneous factors. The point, however, is that correlation was not strengthened by replacing DW with intensity, measured on the usual scale of 1–3, in an attempt to generate a more nuanced measure [10]. Of course, the insensitivity and subjectivity of this scale have already been noted, but, interestingly, Gil-Gouveia et al. found that “HG had no correlation with hours of work lost to headache in the last week” [7].

HG also imports TIS on an ordinal scale of attack duration (“short-lasting < 2 h”, “half-day lasting”, “full-day lasting” or “overnight/24 h”), which is then converted to a numerical scale 1–4 [7], and not as a continuous measure, which obviously it could be. Multiplying this by the disability factor (also 1–4) to generate an *s*_*i*_ of 0–16 [7] treats the numerical TIS scale as an interval scale, which it self-evidently is not. If there is a rationale behind this, it is unclear what it might be, given that HG relies on prospective daily assessment.

And on that last point of prospective daily assessment, much evidence attests that adherence to daily headache diaries is poor [11] (an observation rather than a criticism).

While no single metric can adequately capture the myriad components of headache-attributed burden [12], *time* is of the essence in headache measurement. Whether it is better coupled with some estimate of consequence for greater nuance – symptom intensity, lost health (as in HLDs and HLDs-averted [2], and in GBD’s YLDs [13]), functional impairment (as in HG [6, 7]) or lost productivity (as in HALT [9]) – and, if so, how to do this, remain areas of research of some importance both in the fields of epidemiology, public health and health policy and in clinical management.

## Data Availability

Not applicable.
